# Impact of the COVID-19 pandemic on drug treatment of patients with peripheral arterial disease: an observational cross-sectional study

**DOI:** 10.1590/1677-5449.210021

**Published:** 2021-06-17

**Authors:** Heloisa Amaral Braghieri, Marília de Almeida Correia, Juliana Ferreira de Carvalho, Paulo Longano, Nelson Wolosker, Gabriel Grizzo Cucato, Raphael Mendes Ritti-Dias, Hélcio Kanegusuku

**Affiliations:** 1 Universidade Nove de Julho – UNINOVE, São Paulo, SP, Brasil.; 2 Hospital Israelita Albert Einstein – EINSTEIN, São Paulo, SP, Brasil.; 3 Northumbria University, Newcastle upon Tyne, United Kingdom.

**Keywords:** COVID-19, social isolation, intermittent claudication, drug treatment, health, SARS-CoV-2

## Abstract

**Background:**

The Coronavirus 2019 (COVID-19) pandemic has had a negative impact on the population’s behavior. In this context, the effect of the COVID-19 pandemic on drug treatment of patients with peripheral arterial disease (PAD) and intermittent claudication (IC) remains unclear.

**Objectives:**

To analyze the impact of the COVID-19 pandemic on drug treatment of patients with PAD and IC.

**Methods:**

In this cross-sectional, observational study, 136 patients with PAD and IC were recruited from our database and answered a questionnaire by telephone involving the following questions: a) precautions related to COVID-19; b) general health status; and c) treatment of diseases. Subsequently, patients were divided into two groups according to difficulty in obtaining their drugs (DOD: difficulty obtaining drugs, or NDOD: no difficulty obtaining drugs) and overall health was compared between groups.

**Results:**

Seventeen percent of patients reported difficulties with obtaining drugs during the pandemic. A higher proportion of these patients reported being sadder (56.5% vs. 24.8%, P < 0.01) and having more difficulty sleeping (56.5% vs. 24.8%, P < 0.01) than of the patients in the NDOD group (P <0.01). The groups did not differ in terms of impairment of walking capability, anxiety, stress, or depression (P> 0.05).

**Conclusions:**

A higher proportion of patients in the DOD group reported being sadder and having greater difficulty sleeping compared to the NDOD group during the COVID-19 pandemic.

## INTRODUCTION

Peripheral arterial disease (PAD) has higher prevalence among elderly people and is characterized by stenotic or obstructive lesions of the peripheral arteries that are normally due to atherosclerosis and which reduce or occlude the lumens of vessels and blood flow to the limbs, primarily the lower limbs.^1^^,^^2^ The most prominent risk factors are age, inactivity, diabetes mellitus (DM), systemic arterial hypertension (SAH), dyslipidemia, and smoking.^2^ The reproducible symptoms of intermittent claudication (IC) seen in these patients, characterized by lower limb pain while walking, reduce their physical capability and, as a consequence, exacerbate their comorbidities and significantly reduce their quality of life.^3^^-^6

At the end of 2019, a new coronavirus (SARS-CoV-2) that causes a severe acute respiratory syndrome, named Coronavirus Disease 2019 (COVID-19), was identified for the first time in the city of Wuhan, China, and the subsequent COVID-19 epidemic was declared a global pandemic, with more than 99,363,690 cases and 2,135,950 deaths recorded worldwide by January 26, 2021.^7^ One of the methods for minimizing dissemination of COVID-19, social isolation, has been adopted as an important strategy, particularly for high-risk groups, such as patients with PAD.

Some of the most prominent consequences of the pandemic for populations include increased inactivity and perceived deterioration of physical capability, health, and, as a consequence, quality of life.^8^^-^11 This clinical status can be even more severe for individuals who are exclusively dependent on the public health care system for treatment of diseases, including patients with PAD. These patients have faced difficulties obtaining their medications, since they generally have to collect them in person and need up-to-date medical prescriptions to do so. Social isolation has therefore amplified the treatment difficulties faced by these patients, many of whom were already finding it difficult to correctly adhere to treatment before the Covid-19 pandemic.^12^

In this context, the objective of this study was to analyze the impact of the Covid-19 pandemic on drug treatment of patients with PAD and IC.

## METHODS

### Study design and participants

This was a cross-sectional, comparative, observational study of patients with PAD and IC. Information on the patients’ characteristics was obtained from a study database maintained by the Cardiovascular Disease Clinical Interventions Research and Study Group (GEPICARDIO). Data relating to the impact of COVID-19 on patients were obtained by telephone interviews conducted from May 15 to August 22, 2020, by health professionals with experience in studies with patients with PAD.

This study was approved by the Ethics Committee at the Universidade Nove de Julho (CAAE number 31529220.8.0000.5511; ruling number: 4.023.509). Participants’ responses were included after they had given their consent. All procedures are in compliance with national Brazilian legislation and the Helsinki Declaration.

Patients were included if they met the following criteria: a) PAD diagnosis; b) age ≥ 45 years; c) previous ankle-brachial index ≤ 0.90; d) prior diagnosis of stage II disease according to the Fontaine classification; and e) absence of non-compressible vessels, limb amputations, and/or ulcers. Patients were only excluded if: a) they exhibited some type of deficiency during the phone call that could compromise administration of the questionnaire (e.g. cognitive, auditory, or speech disorders).

### Variables

The personal information accessed from our database were sex (“woman” or “man”), date of birth (DD/MM/AAAA), time since PAD diagnosis (in years), body mass index (kg/m^2^), and severity of PAD (ankle-brachial index and Fontaine stage).^3^^,^^13^ The questionnaire administered is of considerable length, but only the specific questions listed below were employed for the purposes of the present study:

*Smoking:* 1- Do you smoke?; 2- Are you an ex-smoker? The response choices for both items were: “No” or “Yes”.

*Comorbidities*: the participants were read a list of diseases and asked whether they had been diagnosed with each one (diseases: DM, SAH, dyslipidemia, heart disease, respiratory disease, musculoskeletal diseases, or others). The response choices for all items were: “No” or “Yes”:.

*COVID-19*: 1- Are you socially isolating?; 2- Have you been diagnosed with COVID-19?; If yes, 3- Have you recovered? The response choices for all items were: “No” or “Yes”.

*PAD Treatment:* 1- Have you had difficulty obtaining your medications over the last few months because of COVID-19?; 2- Have you had to change the type/dosage of any of your medications?; 3- Are you responsible for collecting your own medications?; 4- How much do you spend per month on medications?; 5- Have you had to cancel an operation? The response options for items 1, 2, 3, and 5 were: “No” or “Yes”. The response options for item 4, were: “Less than R$ 100.00”, “R$ 101 to 200.00”, “R$ 201 to 400.00” or “More than R$ 401.00”.

*Overall health:* 1- How has your health been during the Covid-19 pandemic? (response options: “Good/unchanged” or “Poor”); 2- Have you had difficulty sleeping?; 3- Because of COVID-19, have you been feeling more anxious?; 4- Because of COVID-19, have you been feeling sadder?; 5- Because of COVID-19, have you been feeling more stressed? 6- Because of COVID-19, have you been feeling depressed?; 7- Have you felt that your ability to walk has reduced over the last few weeks? The response options for items 2 to 7 were: “No” or “Yes”.

After the interviews, patients were allocated to one of two groups, depending on whether they had reported difficulty with obtaining their medications (DOD: difficulty obtaining drugs or NDOD: no difficulty obtaining drugs).

### Statistical analysis

All analyses were conducted using the Statistical Package for the Social Sciences^®^ (SPSS, version 20). Comparisons between patients who did and did not encounter difficulty with obtaining their drugs were made using the independent *t* test or the Mann-Whitney U test and chi-square test. Data are expressed as means and standard deviations for continuous variables and relative frequencies for categorical variables. The level of significance was set at p < 0.05.

## RESULTS

**Figure 1:**
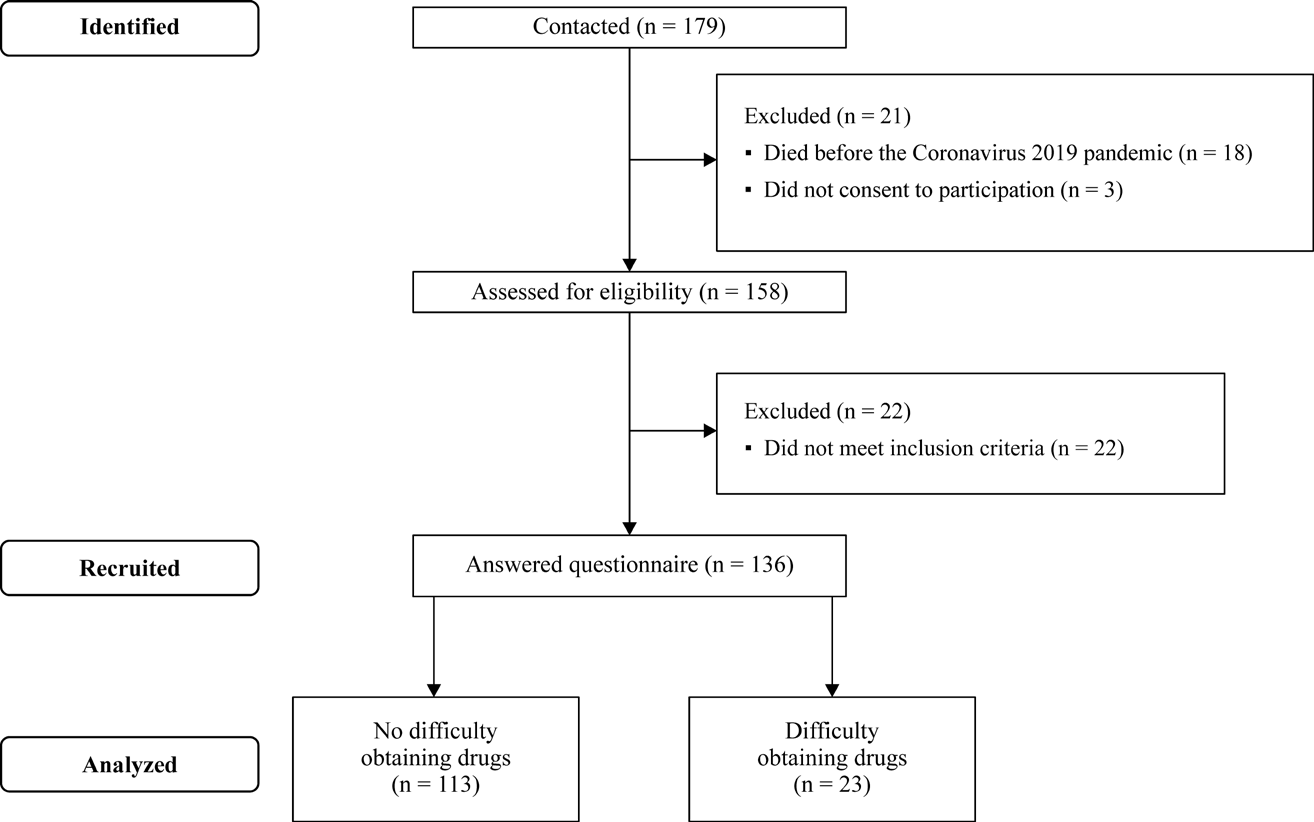
Flow diagram of participants.

A flow diagram illustrating selection and classification of study participants is shown in Figure 1. The 136 patients with PAD and IC who answered the questionnaire were divided into two groups according to whether they had had difficulty with obtaining their drugs (NDOD: 113 patients and DOD: 23 patients). The two groups had similar physical characteristics, risk factors, comorbidities, and percentages of group members practicing social isolation (p > 0.05) (Table 1).

**Table 1:** Characteristics of patients with peripheral arterial disease who did and did not have difficulty obtaining their medications during the COVID-19 pandemic.

Variables	Difficulty obtaining drugs		p
	NDOD (n = 113)	DOD (n = 23)	
Men, %	61.1	60.9	0.99
Age, years	69 ± 9	67 ± 7	0.51
Body mass index, kg/m^2^	27.8 ± 4.3	26.7 ± 3.4	0.28
Ankle-brachial index	0.54 ± 0.18	0.56 ± 0.14	0.66
Time since diagnosis, years#	10 ± 6	10 ± 5	0.43
			
*Risk factors and comorbidities*, %			
Smoker	15.0	17.4	0.78
Ex-smoker	62.8	56.5	0.71
Diabetes mellitus	44.2	47.8	0.75
Hypertension	83.2	87.0	0.65
Dyslipidemia	78.8	69.6	0.34
Obesity	25.7	21.7	0.74
Heart disease	50.4	60.9	0.36
Respiratory disease	14.2	26.1	0.16
Depression	14.2	17.4	0.69
Musculoskeletal diseases	44.2	60.9	0.15
			
Social isolation	88.5	87.0	0.84

Data are expressed as mean ± standard deviation or relative frequency. NDOD = no difficulty obtaining drugs; DOD = difficulty obtaining drugs.

^#^ Mann-Whitney U test p ≤ 0.05.

The groups also had similar results in terms of the percentages of patients responsible for collecting their own medications, the need to change a drug treatment, monthly spending on drugs, and rate of surgery cancellation (p > 0.05) (Table 2).

**Table 2:** Drug treatment and overall health of patients with peripheral arterial disease who did and did not have difficulty obtaining their medications during the COVID-19 pandemic.

Variables	Difficulty obtaining drugs				p
	NDOD (n = 113)		DOD (n = 23)		
*Treatment*	%	95%CI	%	95%CI	
Patient responsible for collecting own medications	34.5	1	39.1	0.5-3.1	0.67
Needed to change type/dosage of a drug	1.8	1	4.5	0.2-29.0	0.44
Monthly spending on medications					
Less than R$ 100.00	72.6	1	52.2	1.0-6.1	0.054
More than R$ 101.00	27.4		47.8		
Cancelled surgery	7.1	1	8.7	0.2-6.3	0.79
					
*Health*					
Overall health					
Unchanged/Good	93.8	1	91.3	0.3-0.7	0.66
Poor	6.2		8.7		
More anxious	47.8	1	65.2	0.8-5.2	0.97
Sadder	24.8	1	56.5*	1.6-10.0	< 0.01
More stressed	24.8	1	26.1	0.4-3.0	0.54
More depressed	21.2	1	13.0	0.2-2.0	0.28
Difficulty sleeping	24.8	1	56.5*	1.6-10.0	< 0.01
Reduced walking capability	44.2	1	39.1	0.3-2.0	0.65

Data are expressed as relative frequency. NDOD = no difficulty obtaining drugs; DOD = difficulty obtaining drugs; CI = confidence interval.

* Significantly different to patients who did not have difficulty obtaining drugs at p ≤ 0.05.

A higher proportion of patients in the DOD group than in the NDOD group reported that they were sadder and had greater difficulty sleeping (p < 0.01). The groups did not differ in terms of overall perceived health, anxiety, stress, depression, or reduced walking capability (p > 0.05) (Table 2).

## DISCUSSION

The main finding of this study was that 17% of the patients reported difficulties obtaining their medications during the pandemic and a greater proportion of these patients reported feeling sadder and having greater difficulty sleeping than those who did not have difficulties obtaining their drugs during the Covid-19 pandemic.

The majority of patients in both groups were elderly, with moderate severity PAD, and had SAH, dyslipidemia, DM, and/or cardiac and musculoskeletal diseases, classifying them as at high risk from COVID-19.^14^^,^^15^ In this respect, 87% of the patients in both groups have been following social isolation recommendations.

However, although social isolation reduces the risk of COVID-19 contagion,^16^^,^^17^ it may also have a negative impact on the drug treatments of some patients with PAD, since they generally collect them in person, for which they need to have up-to-date medical prescriptions, and because many of these patients are dependent on the public healthcare system. In the present study, approximately 17% of the patients reported finding it difficult to obtain their medications during the pandemic. Although we did not observe a difference between the groups in terms of the percentage of patients who stated they were responsible for collecting their own drugs, the patients in the DOD group exhibited a trend to higher monthly spending on medications. In this respect, it could be speculated that these patients’ family incomes could have been impacted by the Covid-19 pandemic. Other factors could also have had an influence on their difficulty obtaining their drugs, such as the regions in which they reside, the regions in which hospitals and health centers are located, and the distance between their places of residence and these services, among other factors. Future studies are needed to investigate this problem.

Social isolation has also had negative impacts on the lifestyle of the population, resulting in worse dietary habits, increased inactivity, and deteriorating health and quality of life.^8^^-^11^,^^18^ This situation may be even more severe among individuals with comorbidities (e.g. patients with psoriasis) with low adherence to drug treatment during the Covid-19 pandemic.^19^ In the present study, a higher proportion of patients with PAD in the DOD group reported feeling sadder and having greater difficulty with sleeping compared with those in the NDOD group. This is of concern, because this scenario has been associated with reduced quality of life in these patients.^20^^-^22

Although there were no differences between the groups in the other health-related parameters, it is important to emphasize that in both groups there was a high prevalence of patients who were more anxious, stressed, and depressed during the Covid-19 pandemic. This result is in line with what has been observed in individuals without PAD.^9^ Patients in both groups also reported deterioration of their capability to walk, which may be related to increased inactivity. Farah et al.^23^ reported an association between inactivity and reduced walking capability in patients with PAD.

Moreover, approximately 8% of these patients had cancelled surgery. This finding is of concern, since Li et al.^24^ observed that the lower number of patients with PAD who underwent surgery during the Covid-19 pandemic had a higher rate of perioperative complications compared with before the pandemic. These results demonstrate the importance of adoption of strategies to improve accessibility to care provided by health professionals and also to make it easier to obtain medications.

This study is subject to certain limitations. 1) it is a cross-sectional study and so cannot establish cause and effect; 2) self-report assessment was employed, making the study susceptible to information bias; 3) the results cannot be extrapolated to other populations with different characteristics; and 4) neither the percentage of patients dependent on the Brazilian National Health Service (Sistema Único de Saúde) who receive their drugs free of charge nor the possible reasons why patients had difficulty obtaining their drugs were investigated.

## CONCLUSIONS

A higher proportion of patients in the DOD group than in the NDOD group reported that they felt sadder and had greater difficulty sleeping during the Covid-19 pandemic.
