# Supplementary material for: Impact of the COVID-19 pandemic on drug treatment of patients with peripheral arterial disease: an observational cross-sectional study
Source: J Vasc Bras. 2021 Jun 17;20:e20210021. doi: 10.1590/1677-5449.210021 (PMC8244965; doi:10.1590/1677-5449.210021)
Supplement: Translation

# Translation: Impacto da pandemia da COVID-19 sobre o tratamento medicamentoso dos pacientes com doença arterial periférica: um estudo observacional transversal

**Authors:** Heloisa Amaral Braghieri, Marília de Almeida Correia, Juliana Ferreira de Carvalho, Paulo Longano, Nelson Wolosker, Gabriel Grizzo Cucato, Raphael Mendes Ritti-Dias, Hélcio Kanegusuku

**Affiliations:** 1 Universidade Nove de Julho – UNINOVE, São Paulo, SP, Brasil.; 2 Hospital Israelita Albert Einstein – EINSTEIN, São Paulo, SP, Brasil.; 3 Northumbria University, Newcastle upon Tyne, United Kingdom.

**Keywords:** COVID-19, isolamento social, claudicação intermitente, tratamento medicamentoso, saúde, SARS-CoV-2

## Abstract

**Contexto:**

A pandemia do Coronavírus 2019 (COVID-19) tem afetado negativamente o comportamento da população. Nesse contexto, o impacto da pandemia da COVID-19 no tratamento medicamentoso dos pacientes com doença arterial periférica (DAP) e claudicação intermitente (CI) permanece obscuro.

**Objetivos:**

Analisar o impacto da pandemia da COVID-19 no tratamento medicamentoso dos pacientes com DAP e CI.

**Métodos:**

Neste estudo observacional transversal, 136 pacientes com DAP e CI, recrutados do nosso banco de dados, responderam por telefone um questionário envolvendo as seguintes questões: a) cuidados com a COVID-19; b) saúde global; c) tratamento das doenças. Posteriormente, os pacientes foram divididos em dois grupos, de acordo com a dificuldade para a aquisição dos medicamentos (DAM: dificuldade e SDAM: sem dificuldade), para a comparação da saúde global entre os dois grupos.

**Resultados:**

Dezessete porcento dos pacientes reportaram dificuldades para a aquisição dos medicamentos durante a pandemia. Uma maior prevalência desses pacientes reportou estar mais triste (56,5% *versus* 24,8%, p < 0,01) e com mais dificuldades para dormir (56,5% *versus* 24,8%, p < 0,01) em relação aos pacientes do grupo SDAM. Os grupos não apresentaram diferenças para o declínio na capacidade de caminhada, ansiedade, estresse e depressão (p > 0,05).

**Conclusões:**

Uma maior prevalência de pacientes do grupo DAM reportou estar mais triste e com mais dificuldade para dormir em comparação ao grupo SDAM durante a pandemia da COVID-19.

## INTRODUÇÃO

A doença arterial periférica (DAP), cuja prevalência é maior entre os indivíduos idosos, é caracterizada pela presença de lesão estenosante ou obstrutiva nas artérias periféricas, normalmente devido à aterosclerose, levando a uma redução ou oclusão na luz do vaso e do fluxo sanguíneo para os membros, principalmente os inferiores^1^^,^^2^. Entre os principais fatores de risco, destacam-se a idade, o sedentarismo, diabetes *mellitus* (DM), hipertensão arterial sistêmica (HAS), dislipidemias e o tabagismo^2^. Nesses pacientes, os sintomas de claudicação intermitente (CI), caracterizada pela dor nos membros inferiores, reprodutível, durante a caminhada, promovem a redução na capacidade física e, consequentemente, piora das comorbidades, impactando significantemente na qualidade de vida desses pacientes^3^^-^6.

No final de 2019, um novo coronavírus (SARS-CoV-2) que causa a síndrome respiratória aguda grave denominada COVID-19, foi identificado pela primeira vez na cidade de Wuhan, China, e, posteriormente, a epidemia da COVID-19 foi declarada uma pandemia global, com mais de 99.363.690 casos e 2.135.950 óbitos registrados em todo o mundo até 26 de janeiro de 2021^7^. Como forma de minimizar a disseminação da COVID-19, o isolamento social tem sido adotado como uma importante estratégia, principalmente entre grupos de risco, como pacientes com DAP.

Entre as consequências que a pandemia trouxe para a população destacam-se o aumento do sedentarismo, percepção de piora na capacidade física, na saúde e, consequentemente, a redução da qualidade de vida^8^^-^11. O quadro clínico pode ser ainda mais grave nos indivíduos que dependem exclusivamente do sistema público de saúde para o tratamento das doenças, incluindo os pacientes com DAP. De fato, esses pacientes têm enfrentado dificuldades para ter acesso aos seus medicamentos, visto que sua obtenção, geralmente, ocorre de forma presencial e em posse de receitas médicas atualizadas. Dessa forma, o isolamento social tem potencializado a dificuldade no tratamento desses pacientes, que comumente já apresentavam dificuldades de segui-lo corretamente antes da pandemia da COVID-19^12^.

Nesse contexto, o objetivo deste estudo foi analisar o impacto da pandemia da COVID-19 no tratamento medicamentoso dos pacientes com DAP e CI.

## MÉTODOS

### Desenho do estudo e participantes

Trata-se de um estudo observacional transversal comparativo, que envolveu pacientes com DAP e CI. As informações das características dos pacientes foram obtidas a partir do banco de dados dos estudos desenvolvidos pelo Grupo de Estudo e Pesquisa em Intervenções Clínicas nas Doenças Cardiovasculares (GEPICARDIO). Os dados relativos ao impacto da COVID-19 nos pacientes foram obtidos por meio de entrevista telefônica, entre 15 de maio a 22 de agosto de 2020, por profissionais de saúde com experiência em estudos com pacientes com DAP.

Este estudo foi aprovado pelo Comitê de Ética da Universidade Nove de Julho (CAAE nº 31529220.8.0000.5511; número do parecer: 4.023.509). As respostas dos participantes só foram incluídas após o consentimento dos mesmos. Todos os procedimentos seguiram a legislação nacional e a Declaração de Helsinque.

Os pacientes foram incluídos se atendessem aos seguintes critérios: a) diagnóstico de DAP; b) idade ≥ 45 anos; c) valor prévio do índice tornozelo-braquial ≤ 0,90; d) diagnóstico prévio com estágio II da classificação de Fontaine; e) ausência de vasos não compressíveis, membros amputados e/ou úlceras. Os pacientes só foram excluídos se: a) apresentassem algum comprometimento durante a ligação telefônica, que pudesse comprometer a aplicação do questionário (e.g. cognitivo, auditivo e de fala).

### Variáveis

As informações pessoais foram acessadas no nosso banco de dados: sexo (“mulher” ou “homem”), data de nascimento (DD/MM/AAAA), tempo de diagnóstico de DAP (em anos), índice de massa corporal (kg/m^2^) e a severidade da DAP (Índice tornozelo-braquial e estágio de Fontaine)^3^^,^^13^. O questionário aplicado é extenso, mas para os objetivos deste estudo foram utilizadas apenas as questões específicas descritas abaixo:

*Tabagismo:* 1- Você fuma?; 2- Você é ex-fumante? As respostas possíveis para todos os itens foram: “Não” ou “Sim”.

*Doenças associadas*: a partir de uma lista de doenças, o participante informou todas as doenças diagnosticadas (i.e. respostas possíveis: DM, HAS, dislipidemias, doença cardíaca, respiratória, osteomuscular ou outros). As respostas possíveis para todos os itens foram: “Não” ou “Sim”.

*COVID-19*: 1- Você está em isolamento social?; 2- Você foi diagnosticado com COVID-19?; Se sim, 3- Você já está recuperado? As respostas possíveis para todos os itens foram: “Não” ou “Sim”.

*Tratamento da doença:* 1- Teve dificuldade para adquirir os seus medicamentos nos últimos meses devido à COVID-19?; 2- Precisou mudar o tipo/dose de algum medicamento?; 3- O senhor é responsável por buscar os seus medicamentos?; 4- Qual o gasto mensal com medicamentos?; 5- Precisou cancelar alguma cirurgia? As respostas possíveis para os itens 1, 2, 3 e 5 foram: “Não” ou “Sim”. Para o item 4, foram: “Até R$ 100,00” ou “R$ 101 a 200,00” ou “R$ 201 a 400,00” ou “Mais de R$ 401,00”.

*Saúde global:* 1- Como está a sua saúde durante a pandemia da COVID-19? (respostas possíveis: “Boa/igual” ou “Ruim”); 2- Tem sentido dificuldade para dormir?; 3- Devido à COVID-19, você está se sentindo mais ansioso?; 4- Devido à COVID-19, você está se sentindo mais triste?; 5- Devido à COVID-19, você está se sentindo mais estressado? 6- Devido à COVID-19, você está se sentindo deprimido?; 7- Você sentiu que a sua capacidade de caminhada diminuiu nas últimas semanas? As respostas possíveis para as questões 2 a 7 foram: “Não” ou “Sim”.

Ao final das entrevistas, os pacientes foram divididos em dois grupos, de acordo com a dificuldade para a aquisição do medicamento (DAM: dificuldade para adquirir o medicamento e SDAM: sem dificuldade para adquirir o medicamento).

### Análise estatística

Todas as análises foram realizadas pelo *software* Statistical Package for the Social Sciences® (SPSS, versão 20). A comparação entre os pacientes que tiveram e os que não tiveram dificuldades para adquirir os medicamentos foi realizada através do teste *t* independente ou teste U de Mann-Whitney e o teste de qui-quadrado. Os dados estão apresentados como média e desvio padrão para as variáveis contínuas e frequência relativa para as variáveis categóricas. A significância foi definida com p < 0,05.

## RESULTADOS

Um fluxograma do estudo é fornecido na Figura 1. Os 136 pacientes com DAP e CI que responderam ao questionário foram divididos em dois grupos, de acordo com a dificuldade para a aquisição dos medicamentos (SDAM: 113 pacientes e DAM: 23 pacientes). Os grupos apresentaram semelhantes características físicas, fatores de risco e doenças associadas e porcentagem de pessoas seguindo o isolamento social (p > 0,05) (Tabela 1).

**Figura 1:** Fluxograma dos participantes.

**Tabela 1:** Características dos pacientes com doença arterial periférica que não tiveram e que tiveram dificuldades para adquirir os medicamentos durante a pandemia de COVID-19.

Variáveis	Dificuldade para adquirir os medicamentos		p
	SDAM (n = 113)	DAM (n = 23)	
Homens, %	61,1	60,9	0,99
Idade, anos	69 ± 9	67 ± 7	0,51
Índice de massa corporal, kg/m^2^	27,8 ± 4,3	26,7 ± 3,4	0,28
Índice tornozelo-braquial	0,54 ± 0,18	0,56 ± 0,14	0,66
Tempo de diagnóstico, anos#	10 ± 6	10 ± 5	0,43
			
*Fatores de risco e doenças associadas*, %			
Fumante	15,0	17,4	0,78
Ex-fumante	62,8	56,5	0,71
Diabetes *mellitus*	44,2	47,8	0,75
Hipertensão	83,2	87,0	0,65
Dislipidemia	78,8	69,6	0,34
Obesidade	25,7	21,7	0,74
Doença cardíaca	50,4	60,9	0,36
Doença respiratória	14,2	26,1	0,16
Depressão	14,2	17,4	0,69
Doença osteomuscular	44,2	60,9	0,15
			
Isolamento social	88,5	87,0	0,84

Dados estão apresentandos como média ± desvio padrão e como frequência relativa. SDAM = sem dificuldade para adquirir os medicamentos; DAM = com dificuldade para adquirir os medicamentos.

^#^ Teste U de Mann-Whitney. p ≤ 0,05.

Os grupos também apresentaram resultados similares em relação à porcentagem de pacientes responsáveis por buscarem os próprios medicamentos, necessidade de mudança no tratamento medicamentoso, gasto mensal com os mesmos e taxa de cancelamento das cirurgias (p > 0,05) (Tabela 2).

**Tabela 2:** Tratamento medicamentoso e a saúde global dos pacientes com doença arterial periférica que tiveram e que não tiveram dificuldade para adquirir os medicamentos durante a pandemia de COVID-19.

Variavéis	Dificuldade para adquirir os medicamentos				p
	SDAM (n = 113)		DAM (n = 23)		
*Tratamento*	%	IC95%	%	IC95%	
Responsável por buscar os medicamentos	34,5	1	39,1	0,5-3,1	0,67
Precisou mudar o tipo/dose de algum medicamento	1,8	1	4,5	0,2-29,0	0,44
Gasto mensal com medicamentos					
Até R$ 100,00	72,6	1	52,2	1,0-6,1	0,054
Mais de R$ 101,00	27,4		47,8		
Cancelou alguma cirurgia	7,1	1	8,7	0,2-6,3	0,79
					
*Saúde*					
Saúde global					
Igual/Boa	93,8	1	91,3	0,3-0,7	0,66
Ruim	6,2		8,7		
Mais ansioso	47,8	1	65,2	0,8-5,2	0,97
Mais triste	24,8	1	56,5*	1,6-10,0	< 0,01
Mais estressado	24,8	1	26,1	0,4-3,0	0,54
Mais deprimido	21,2	1	13,0	0,2-2,0	0,28
Dificuldade para dormir	24,8	1	56,5*	1,6-10,0	< 0,01
Declínio na capacidade de caminhada	44,2	1	39,1	0,3-2,0	0,65

Dados estão apresentados como frequência relativa. SADM = sem dificuldade para adquirir os medicamentos; DAM = com dificuldade para adquirir os medicamentos; IC = intervalo de confiança.

* Significante diferente dos pacientes que não tiveram dificuldades para adquirir os medicamentos. p ≤ 0,05.

Uma maior prevalência de pacientes do grupo DAM reportou estar mais triste e com mais dificuldades para dormir em relação aos pacientes do grupo SDAM (p < 0,01). Os grupos não apresentaram diferenças para a percepção da saúde geral, ansiedade, estresse, depressão e declínio na capacidade de caminhada (p > 0,05) (Tabela 2).

## DISCUSSÃO

O principal achado do presente estudo foi que 17% dos pacientes reportaram dificuldades para a aquisição dos medicamentos durante a pandemia e uma maior prevalência desses pacientes reportou estar mais triste e com mais dificuldade para dormir em comparação ao grupo SADM durante a pandemia da COVID-19.

A maioria dos pacientes de ambos os grupos é de idosos, com moderada severidade da DAP e apresentam HAS, dislipidemias, DM e doenças cardíacas e osteomusculares, o que os classifica como grupo de risco para a COVID-19^14^^,^^15^. Nesse contexto, 87% dos pacientes de ambos os grupos têm seguido as recomendações para o isolamento social.

No entanto, apesar do menor risco de contágio da COVID-19 com a adoção do isolamento social^16^^,^^17^, isso também pode estar impactando negativamente no tratamento medicamentoso de alguns pacientes com DAP, visto que a sua obtenção geralmente ocorre de forma presencial e em posse de receitas médicas atualizadas, sendo que muitos pacientes são dependentes do sistema público de saúde. No presente estudo, aproximadamente 17% dos pacientes reportaram dificuldades para a aquisição dos medicamentos durante a pandemia. Embora não tenhamos observado diferenças entre os grupos na porcentagem de pacientes que reportaram ser responsáveis por buscar o próprio medicamento, os pacientes do grupo DAM apresentaram uma tendência a ter um maior gasto mensal com os medicamentos. Nesse contexto, é possível especular que a renda familiar desses pacientes tenha sido afetada durante a pandemia da COVID-19. Outros fatores também podem ter influenciado na dificuldade da aquisição dos mesmos, como a região onde se encontram os hospitais e os postos de saúde, a distância da residência e esses lugares, entre outros. Futuros estudos são necessários para investigar essa problemática.

O isolamento social também tem impactado negativamente no estilo de vida da população, resultando em um pior hábito alimentar, aumento do sedentarismo e piora da saúde e da qualidade de vida^8^^-^11^,^^18^. Esse quadro pode ser ainda mais grave entre os indivíduos portadores de comorbidades (e.g. pacientes com psoríase) com baixa aderência ao tratamento medicamentoso durante a pandemia da COVID-19^19^. No presente estudo, uma maior prevalência dos pacientes com DAP do grupo DAM reportaram estar mais triste e com mais dificuldade para dormir em comparação ao grupo SDAM. Isso é preocupante, pois esse quadro tem sido associado à uma redução na qualidade de vida desses pacientes^20^^-^22.

Embora os outros parâmetros relacionados à saúde não tenham apresentado diferenças entre os grupos, é importante destacar que uma alta prevalência dos pacientes em ambos os grupos está mais ansiosa, estressada e deprimida durante a pandemia da COVID-19. Esse resultado está de acordo com o observado em indivíduos sem a DAP^9^. Os pacientes de ambos os grupos também reportaram prejuízo na capacidade de caminhada, o que pode estar relacionado com o aumento do sedentarismo. Farah et al.^23^ reportaram associação entre sedentarismo e a pior capacidade de caminhada em pacientes com DAP.

Ademais, aproximadamente 8% desses pacientes cancelaram as cirurgias. O quadro é preocupante, uma vez que Li et al.^24^ observaram que o número reduzido de pacientes com DAP submetidos a cirurgias durante a pandemia da COVID-19 apresentavam uma maior taxa de complicações perioperatórias comparada aos de antes da pandemia. Esses resultados demonstram a importância da adoção de estratégias que tornem mais acessíveis os atendimentos com os diferentes profissionais da área da saúde, assim como uma maior facilidade na aquisição dos medicamentos.

Este estudo possui algumas limitações. 1) é um estudo transversal e não permite estabelecer causa e efeito; 2) foi utilizada uma avaliação autorreferida, o que torna suscetível o viés de informação; 3) os resultados não podem ser extrapolados para outras populações com outras características; 4) não foi avaliada a porcentagem de pacientes dependentes do Sistema Único de Saúde e que recebem gratuitamente os medicamentos para o tratamento da DAP, assim como os possíveis motivos referentes à dificuldade para a obtenção dos mesmos.

## CONCLUSÃO

Uma maior prevalência de pacientes do grupo DAM reportou estar mais triste e com mais dificuldade para dormir em comparação ao grupo SDAM durante a pandemia da COVID-19.
